# Road traffic injuries in Kenya: a survey of commercial motorcycle drivers

**DOI:** 10.11604/pamj.2015.21.17.5646

**Published:** 2015-05-07

**Authors:** Duncan Mwangangi Matheka, Faraj Alkizim Omar, Chebiwot Kipsaina, Jeffrey Witte

**Affiliations:** 1 Department of Surgery, Machakos Level 5 Hospital, Machakos, Kenya; 2 Department of Medical Physiology, University of Nairobi, Nairobi, Kenya; 3 AMEND, New York, USA; 4 Centres for Health and Education Programmes, Nairobi, Kenya; 5 Department of Forensic Medicine, Monash University, Melbourne, Australia

**Keywords:** Africa, boda-boda, negligence, speeding, young males

## Abstract

**Introduction:**

Motorcycle injuries contribute a substantial number of deaths and hospital admissions in Kenya. There is paucity of data to inform prevention strategies to address the issue. Therefore, the current study sought to explore the characteristics of 2 and 3-wheeler related road traffic injuries (RTIs) in Kenya.

**Methods:**

A cross-sectional survey of motorcycle drivers involved in a RTI in the preceding 3 months was conducted in 11 urban and rural sites in Kenya's Thika town through face- to -face structured interviews. Drivers’ demographic information, comprehensive crash characteristics and socioeconomic impact of injury data were collected.

**Results:**

Of 200 drivers injured, 98% were male, with average age of 28.4 years (SD±6.6). Of these drivers, 33% were not wearing any protective equipment. Negligence was the most reported cause of crash (33%), followed by slippery roads (21.0%) and speeding (17.5%). The risk of sustaining a bodily injury was 1.3 times higher in drivers who had not received prevention education compared to those who had received such education. People injured at night were 5 times more likely to sustain a bodily injury compared to those injured during the day. Only 8.5% of the drivers reported the injury incident to the police.

**Conclusion:**

Majority of motorcycle related injuries in Thika town occur among young, productive, working-age male drivers. A high proportion of injuries are due to negligence on riding while not wearing any protective equipment compounded by lack of injury prevention education. Initiatives to foster helmet wearing, provision of high-quality affordable helmets, responsible driving and advocacy for stronger legislation, are recommended.

## Introduction

Road traffic injuries (RTIs) account for nearly 1.24 million deaths each year, with another 20-50 million sustaining a form of minor to major road traffic related injury [1, 2]. The African region has the highest road fatality rates globally (24.1 deaths per 100,000 population), well above the global average of 18.0 deaths per 100,000, in spite of the fact that the region is the least motorized with 2% of the world's vehicles, of the six World Health Organization (WHO) regions [3]. Kenya, a low income country in East Africa, has an estimated road fatality rate of 20.9 per 100,000 population [3], higher than that of the European region (10.3 per 100,000 population) [3, 4]. The Kenyan Vision 2030, launched in the year 2008, aims to transform Kenya into a middle-income country from the current third world state [5]. Working towards achieving this, the Kenya government recognizes that transportation is one of the key pillars in achieving this strategy, and has therefore invested not only in improving the physical road infrastructure but also prioritizing road safety as an urgent systemic issue. In 2012, National Transport and Safety Authority (NTSA) was founded as the lead authority in Kenya for road transport and safety [6]. The organization recognizes motorcycle operation as a big challenge in curbing transport related injuries in Kenya [6].

In 2010, according to the Kenya Traffic Police Department, there were 3,055 road traffic related deaths in Kenya, though this is likely to be an underestimate [7]. According to official statistics, 54 school children were killed, 165 seriously injured and 49 sustained minor injuries in the year 2012 around Thika town, where the current study was conducted. All these children were knocked down by motorists and cyclists while crossing roads to and from school [8]. A similar picture is reflected across the country, where in 2013 the NTSA reported that 13,028 people were involved in road crashes. The social and economic costs to the country are huge, with NTSA estimating that RTIs cost Kenya's economy approximately Ksh 14 billion (5% of the country's Gross Domestic Product) [6]. Most incidents in Kenya (62%) occur among the vulnerable road users including pedestrians, cyclists and 2 or 3 wheeler drivers [7], a similar pattern found in other African countries such as Tanzania, Nigeria and Ghana [9–12]. The increase in use of motorised 2 or 3 wheelers as forms of transport have been attributed to their accessibility, affordability and an unregulated market in developing countries including Kenya [12, 13].

Currently there is no official data on total number of registered motorcycles, though other sources estimate that motorcycles account for 70% of motor vehicles registered in Kenya each year [3]. In Kenya, to legally drive a motorcycle, one must be aged 18 years or older, possess a driving licence, and wear a helmet and reflective clothing while riding. However, enforcement of the national motorcycle safety law has been inefficient. RTIs are a multifactorial issue and high quality data for decision making is integral to the equation. The lack of useful, reliable and timely data to inform prevention strategies has been one of the major contributing factors hindering Kenya's efforts to address motorcycle related injuries through policy and legislative responses within risk groups. One relevant function and mandate of the NTSA is to conduct research and audits on road safety to inform road safety strategies [6]. In promoting and working in line to achieving this, the current research study was conducted aimed to understand the trends, patterns and risk factors associated with RTIs among the vulnerable road users in order to reduce the rising RTI incidence in Kenya.

## Methods

**Study design and setting:** A cross-sectional descriptive survey of commercial motorcycle taxis was conducted in January and February 2014 at Thika town (Central Kenya) and its surroundings. Thika town is located approximately 50 km North of Nairobi with a population of 650,000 [14]. It is a fast growing town especially since the opening of the Nairobi-Thika Superhighway (a state-of the art busy road with 8-12 lanes) in 2012 and identified by the WHO as one of the high impact roads in the country, where 80% of victims presenting to the nearby district hospitals sustain their injuries [15]. Eleven sites were selected conveniently where bicycle, motorcycle and three wheeler auto rickshaw drivers were interviewed. These sites were selected with the support of local authorities, three of which were within Thika's central business district, and the remaining were in residential areas.

**Data collection:** An inclusion criteria of having been involved in a road traffic crash within the preceding 3 months was applied. Data collected included demographic information, details on their crash, as well as the RTI impact on socioeconomic status. Data were collected through face- to face interview using a structured questionnaire.

**Ethics approval:** Approval was obtained from the Thika Traffic Police Base Commandant and the Thika boda-boda association. All information obtained was confidential and used for the sole purpose of the study. The study protocol was explained to each of the drivers, and a written consent was obtained from each prior to interview. No identifying data was collected from the participants.

**Data management:** Data were cleaned in Microsoft Excel and transferred into Statistical Package for Social Sciences (SPSS) version 17.0 (SPSS Inc., Chicago, Illinois) for analysis. Descriptive data were summarized and reported.

## Results

### Demographic characteristics

Five hundred and twenty eight drivers agreed to participate in the study. Of these, a total number of 200 drivers met the inclusion criteria, and were interviewed. Their average age was 28.4 years (SD±6.6) with people aged 18 to 24 years and 25 to 31 years accounting for 29% and 47% of those injured, respectively. Of these 200 drivers, 98% were male, 2% were female giving a ratio of 49:1 respectively (Table 1).

**Table 1:** Characteristics of study population by age and sex

Age Category (years)	Sex					
	Male		Female		Total	
	No	%	No	%	No	Total%
18-24	54	27.7	3	60.0	57	28.5
25-31	92	47.2	2	40.0	94	47.0
32-38	34	17.4	0	0.0	34	17.0
39-45	10	5.1	0	0.0	10	5.0
46-54	5	2.6	0	0.0	5	2.5
Total	195	100	5	100	200	100

### Type of vehicle and time of injury

Of the 200 respondents, majority 123 (61.5%) sustained an injury while riding a motorcycle followed by bicycle riders 71(35.5%). Most crashes 64 (32.0%) occurred during daytime (Table 2). The study found that people injured in the night were 5 times more likely to sustain a bodily injury compared to daytime (Unadjusted OR 5.3, 95% CI 1.7- 16.2, p=0.00).

**Table 2:** Characteristics of injures among the 200 two-wheeler drivers

Characteristics	No. of drivers (N=200)	%
**Taxi**		
Motor cycles	123	61.5
Auto rickshaws	6	3
Bicycles	71	35.5
**Activity at time of injury**		
Work related	187	93.5
Non-work related	13	6.5
**Type of road**		
Highway	46	23
Paved non-highway	77	38.5
Gravel	15	7.5
Dirt road	61	30.5
Parking lot	1	0.5
**Time of day injury occurring**		
Morning	44	22
Daytime	64	32
Sunset	58	29
Night	34	17
**Type of injury**		
Cut	29	14.5
Bruise	72	36
Fracture/dislocation	22	11
Concussion	3	1.5
Minor and None	74	38
**Medical attention sought**		
Yes	101	50.5
No	99	49.5
**Length of stay in hospital (Nights)**		
Total	216	
Mean	2.2	

### Scene of injury

Majority of the crashes, 77 (38.5%), occurred on paved non-highway roads while 61 (30.5%) occurred on dirt road and 46 (23%) occurred on main highway. Of the 123 motorcycle riders, 45 (36.6%) of the injuries occurred on paved non-highway road with 39 (31.7%) occurring on a dirt road (Table 3).

**Table 3:** Place of occurrence of motorcycle crash by type of road and type of 2-wheeler vehicle

Type of road	Boda-boda	Three heeler	Bicycle	Total
Highway	27 (22.0)	0 (0.0)	19 (26.8)	46 (23.0)
Paved non-highway	45 (36.6)	5 (83.3)	27 (38.0)	77 (38.5)
Gravel	11 (8.9)	1(16.7)	3 (4.2)	15 (7.5)
Dirt road	39 (31.7)	0(0.0)	22 (31.0)	61(30.9)
Parking lot	1 (0.8)	0 (0.0)	0 (0.0)	1 (0.5)
Total	123(100.0)	6 (100.0)	71 (100.0)	200 (100.)

### Reported types and causes of injury

**Figure 1:**
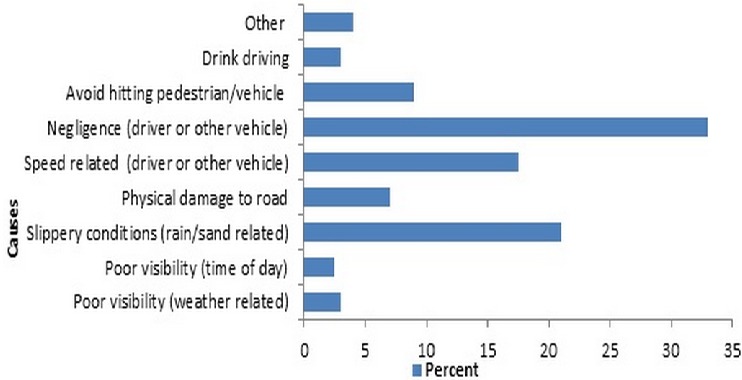
Reported causes of crash

Minor injuries (38.0%) and bruises (36.0%) accounted for a greater part of the injuries among the drivers (Table 2, Table 3). Negligence of the drivers was the most reported cause of crashes (33.0%), followed by slippery conditions caused by rain or sand (21.0%). Speeding (17.5%) and swerving to avoid hitting pedestrian or other vehicles (9%) were other important causes identified (Figure 1). Only 8.5% of the drivers reported the injury incident to the police. Of the 200 respondents, 126 (68.8%) reported to have lost some income following the injury.

### Safety precaution measures

**Figure 2:**
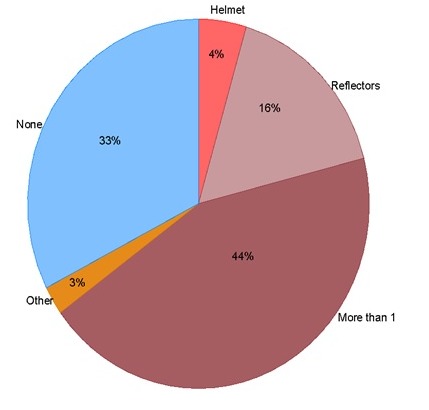
Proportions of protective equipment utilized by drivers during time of crash

Most of the respondents (44%) reported to have been wearing more than one protective gear, while 4% wore a helmet only and 16% wore reflective clothing only, at the time of crash. However, at the time of crash, 33% of the respondents were not wearing any protective equipment (Figure 2). The odds of an injury in those that had protective gear were 27% less than those not wearing any protection. Furthermore, unadjusted analysis showed that the risk of sustaining a bodily injury was 1.3 times higher (95% CI 0.7-2.4, p=0.43) in those respondents who had not received some prevention education compared to those who had received some form of prevention education.

## Discussion

During the study period, a total of 200 two- and three- wheeler vehicle drivers who had sustained a crash in the preceding 3 months were interviewed on the characteristics, socioeconomic impact and details surrounding the crashes. The crashes predominantly involved young working-age males (98%); with a substantially high proportion being negligent in the road (33%), with no injury prevention education, and not wearing any protective clothing (33%). This study highlights that majority of the motorcycle related injuries occur among young aged working male motorcycle drivers. This is comparable to other studies[9–12], and the WHO report which indicate that among persons aged between 15 and 29 years, RTIs are the leading cause of mortality [16].

In Kenya, often young men are the ones predominantly involved in the motorcycle business, yet they are poorly trained, often unlicensed and inexperienced motorcyclists and more likely to take risks [17]. The outcomes of such injuries have an impact on a country, not only on its working population but also on families where most of the drivers are the family bread winners as exemplified by the current study where 70% of the injured reported to have lost some income following the injury. This also has a spill over effect on already overstretched healthcare system and country's economy. Motorcycle taxis accounted for most of the crashes (65%) in the current study. There are several factors that could be attributed to this; firstly, relative affordability and un-regulated importation have seen an influx of motorcycles in Kenya making it a popular means of affordable mode of transport [3, 12]. Secondly, this can be ascribed to Kenya's incomprehensive legislation to curb most of the risk factors such as drunk driving, speeding, failure to use protective gear (such as helmets, seat belts and child restraints) [15]. A high number of the drivers (33%) were not wearing any protective equipment while riding, yet there is established evidence showing that helmet wearing reduces the risk of head injury by 69% and death by 42% [18]. Among the crash victims, very few sought healthcare services and majority opted for over the counter medication. Only 8.5% of the boda-boda drivers reported the injury incident to the police. This also demonstrated a neglected aspect of road safety in Kenya - post RTI care. A survey done by the WHO in 2013, showed that there was no comprehensive emergency surveillance system and no specific emergency contact number available for RTI victims [3]. Such victims are therefore more likely to have delayed access to medical care.

### Study limitations

This study was not without limitations: This was a convenience cross-sectional study conducted at a single town in Kenya and therefore the results may not be generalized to the rest of the country. Data collected was self-reported hence the results may have been biased since the accuracy of respondents’ description on circumstances of the injury could not be independently verified. As some participants may have reported injury events that may have occurred outside the study period, there is a possibility that some details of the injury event may have been missed, hence introducing recall bias to the study. The short recall period of 3 months was made in order to minimize this, however it is also acknowledged that the short period of time for data collection that focused on only RTIs within the preceding 3 months, did not take into consideration the seasonality changes that could affect RTIs through the year. We therefore recommend conducting of a more robust study to not only quantify the issue but to understand and ascertain why drivers are not wearing helmets or any protective equipment while riding.

Minor injuries and bruises accounted for majority of the injuries among the drivers. This is however not representative of all motorcycle related injuries as the cohort interviewed were still working as motorcycle drivers leaving out victims of more severe injuries that may have been forced to abandon the occupation. Studies have reported that the common injuries sustained in RTI in Kenya include limb fractures accounting for 27% and head injuries accounting for 25% [19].

## Conclusion

Majority of motorcycle related injuries occur among young, productive, male drivers who are mostly negligent on riding while not wearing any protective equipment compounded by lack of injury prevention education. It is therefore imperative to advocate for road safety measures such as safe, responsible and disciplined driving, as well as advocate for provision and utilization of safety gear including high-quality affordable helmets, reflective jackets and paddings. Strict legislation and enforcement of road safety laws are necessary to reduce RTI related morbidity and mortality.
